# CXCR2 Mediates Distinct Neutrophil Behavior in Brain Metastatic Breast Tumor

**DOI:** 10.3390/cancers14030515

**Published:** 2022-01-20

**Authors:** Simrit Safarulla, Ankit Madan, Fei Xing, Arvind Chandrasekaran

**Affiliations:** 1 Bioinspired Microengineering Laboratory (BIOME), Department of Chemical, Biological and Bioengineering, NC A&T State University, Greensboro, NC 27411, USA; sarakkal@aggies.ncat.edu; 2 Department of Internal Medicine, SOVAH Cancer Center, Danville, VA 24541, USA; Ankit.Madan@LPNT.net; 3 Joint Department of Cancer Biology, Wake-Forest University School of Medicine, Winston-Salem, NC 27157, USA; fxing@wakehealth.edu

**Keywords:** neutrophils, Neutrophil Extracellular Traps (NETs), CXCR2, chemotaxis, apoptosis, Tumor-Associated Neutrophils (TANs), breast cancer, brain metastasis, AZD5069, Neutrophil Elastase Inhibitor

## Abstract

**Simple Summary:**

Brain metastasis is one of the common complications associated with breast cancers. Neutrophils form the predominant type of circulating white blood cells and play an important role in tumor pathogenesis. However, the role of neutrophils in the evolution of brain metastasis of breast cancers has not been clearly understood. Using simple neutrophil-tumor cell-culture models, we studied the possible biomolecular mechanisms by which the brain-metastatic breast cancer cells could potentially re-program the neutrophils as a tumor-promoting strategy, and how drug-induced inhibition of certain key neutrophil functionalities could help reverse this behavior.

**Abstract:**

Brain metastasis is one of the main causes of mortality among breast cancer patients, but the origins and the mechanisms that drive this process remain poorly understood. Here, we report that the upregulation of certain CXCR2-associated ligands in the brain metastatic variants of the breast cancer cells (BrM) dynamically activate the corresponding CXCR2 receptors on the neutrophils, thereby resulting in the modulation of certain key functional neutrophil responses towards the BrM. Using established neutrophil-tumor biomimetic co-culture models, we show that the upregulation of CXCR2 increases the recruitment of Tumor-Associated Neutrophils (TANs) towards the BrM, to enable location-favored formation of Neutrophil Extracellular Traps (NETs). Inhibition of CXCR2 using small molecule antagonist AZD5069 reversed this behavior, limiting the neutrophil responses to the BrM and retarding the reciprocal tumor development. We further demonstrate that abrogation of NETs formation using Neutrophil Elastase Inhibitor (NEI) significantly decreases the influx of neutrophils towards BrM but not to their parental tumor, suggesting that CXCR2 activation could be used by the brain metastatic tumors as a mechanism to program the tumor-infiltrating TANs into a pro-NETotic state, so as to assume a unique spatial distribution that assists in the subsequent migration and invasion of the metastatic tumor cells. This new perspective indicates that CXCR2 is a critical target for suppressing neutrophilic inflammation in brain metastasis.

## 1. Introduction

The brain is considered to be one of the most common organs of hematogenous tumor metastasis, accounting for ~30% of all cancer metastases [1,2,3,4]. In breast cancers, the risk of brain metastasis varies differently among hormone positive (~14%), Human epidermal growth factor receptor-2 (HER2) positive (~34%), and triple negative (~46%) receptor subtypes [5,6,7,8]. However, brain metastasis continues to remain the most intractable issue for patients suffering from metastatic breast cancers [9,10], with limited treatment options and consequently, poor survival (7~9 months). Whereas the choice of systemic antitumor therapy relies heavily on receptor status studies conducted with biopsies of the metastatic lesions, brain metastases biopsies are not routinely performed due to the morbidity of the procedure, consequent to the availability of targeted agents and status of the extracranial disease at the time of diagnosis. Therefore, understanding the originating biomolecular and cellular mechanisms of the modalities by which brain metastasis could evolve from the parental breast tumor is critical for offering better assistance towards the development of potential diagnostic or therapeutic targets, and innovative treatment paradigms [11].

Neutrophils, comprising up to 50–60% of the circulating white blood cells, are one of the most abundant immune cell types present within a Tumor-Immune Microenvironment (TIME) [12,13,14]. They are increasingly being recognized as the critical tumor-infiltrating immune cells [15,16,17,18,19] that have a unique influence on the tumor initiation, development and response to therapies [12,13,14]. Though traditionally neutrophils were considered to possess an innate ability to retard tumor growth [20,21,22], in general, multiple studies also support the notion that neutrophils and their pro-tumorigenic subtypes [23,24,25] could promote tumor metastasis by regulation of tumor survival, reawakening of dormant tumor cells, tumor migration and invasion, and modulation of immune response [26,27,28,29,30,31,32,33,34,35,36,37]. Under certain conditions, neutrophils respond to pro-inflammatory stimuli by forming web-like structures composed of histones and decondensed chromatin, collectively termed as Neutrophil Extracellular Traps (NETs) [38]. These NETs could aid metastatic seeding of tumor cells and colonization of the seeded tumor cells in host organs [39,40,41]. Recent reports suggest that cancer cells can also induce NETs to support tumor progression and metastasis [12,42,43,44,45,46,47].

**Figure 1:**
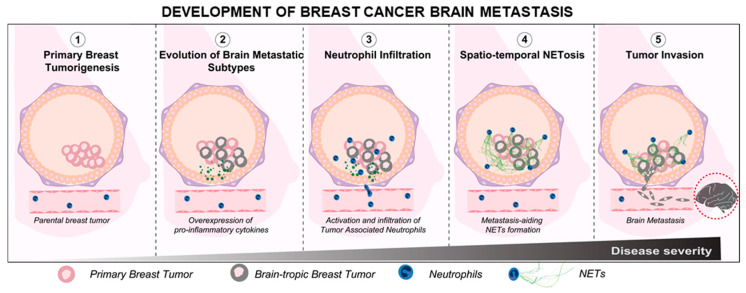
Schematic representation of the breast cancer brain metastasis development and the associated tumor-promoting functionalities of the neutrophils.

In general, higher numbers of neutrophils have been associated with poorer survival outcomes in patients with breast cancer brain metastasis, even after surgical interventions [48]. Whether or not the neutrophils actively orchestrate the brain-tropism of breast cancer metastasis is largely unknown. However, recent studies show that certain neutrophil subtypes could infiltrate brain metastases to aid tumor development [49]. We further verified this in our preliminary assay, wherein we observed that the infiltrating neutrophils could also respond to brain metastasis through the formation of NETs within the metastatic niche (Supplemental Figure S1). Therefore, based on the available information of neutrophil involvement in breast tumor progression, it is reasonable to hypothesize that the neutrophil infiltration and activation within a primary TIME could, at least in part, mediate the metastatic cascade of the brain-tropic breast tumor cells (schematically shown in Figure 1).

However, a more fundamental question that remains to be answered is: how would the neutrophil activity and plasticity within a primary TIME be regulated differently [50], depending on the existence of intrinsic differences in the inflammatory signals expressed, by the pro-brain metastatic subtypes present within the parental breast cancer (PBC) [51]? Understanding the mechanisms by which brain-tropic metastatic breast cancer (BrM) cells present within a TIME could activate the neutrophils differently, modulate their tumor-infiltration behavior, and regulate their subsequent metastasis-aiding functionalities, including NETosis. We believe this represents an interesting challenge that merits further probing. Therefore, in this work, we conducted an in vitro investigation of the wholesome neutrophil response toward brain-metastasis specific breast cancer cells, in an attempt to elucidate the functional differences in the manner by which the dynamics of a heterogeneous population of neutrophils could be programmed distinctly by brain-tropic breast tumor subtypes available within the parental breast tumor.

## 2. Materials and Methods

### 2.1. Cell Culture 

MDA-MB231 (MDA-231 for brevity), a highly aggressive and metastatic triple negative parental breast tumor cell line, and its brain metastatic subtypes, MDA-MB231BrM2a (abbreviated henceforth as MDA-BrM) were used as the respective tumor cell lines. MDA-231 was purchased from ATCC and MDA-MB231BrM2a (MDA-BrM) was a kind gift from Dr. Joan Massagué (Memorial Sloan-Kettering Cancer Center, New York, NY, USA). Both these cells were cultured in Dulbecco’s Modified Eagle Medium (DMEM) supplemented with 10% Fetal Bovine Serum (FBS) and 1% Antibiotic-Antimycotic. Cells were grown and maintained in a humidified environment with 5% CO_2_ at 37 °C.

To generate tumor conditioned media (TCM), the respective tumor cell lines were cultured at ~80% confluency in T75 tissue culture flasks. The growth media was then removed and incubated with fresh medium for 24 h. The media was collected and centrifuged to remove dead cell debris. Conditioned Media (CM) aliquots were frozen at −20 °C until required (not exceeding 2 weeks).

### 2.2. Isolation of Neutrophils

Neutrophils were isolated from whole blood drawn from healthy anonymous donors by an immunomagnetic negative selection protocol, as described previously [12,52]. First, red blood cells were aggregated and removed from the whole blood, followed by the selective identification and elimination of non-neutrophils using antibody complexes and magnetic particles, leaving the neutrophils unstimulated. The isolated neutrophils were treated with 16.2 µM Hoechst 33,258 dye (H21491, Invitrogen, Waltham, MA, USA) at 37 °C for 15 min for nuclear staining and prepared to the desired final concentration for the experiments in RPMI 1640 medium. As applicable, naive neutrophils were allowed to form NETs by interacting with tumor cells or their conditioned media. To inhibit the NETs formation, neutrophils were pre-treated with 10 µM of Sivelestat (HY-17443, Medchemexpress, South Brunswick Township, NJ, USA), a Neutrophil Elastase Inhibitor, prior to the assays.

Image-iT™ LIVE Green Caspase-3 and -7 Detection Kit (I35106, Invitrogen™, Waltham, MA, USA) was used to detect pro-apoptotic neutrophils. The cells were incubated with 30X fluorescent inhibitor of caspases (FLICA™, Bio-Rad, Hercules, CA, USA) reagent at 37 °C with 5% CO_2_ for 1 h protected from light. The solution was removed and gently rinsed with RPMI 1640 medium. The cells were then washed twice with a 1X apoptosis wash buffer prior to imaging. As required, Annexin-V (A23204, Thermofisher, Waltham, MA, USA) labeling for the apoptosis assays was performed, following the protocol recommended by the manufacturer.

#### 2.2.1. Microfluidic Neutrophil Chemotaxis Assays

To assay directed neutrophil chemotaxis to MDA-BrM and MDA-231, we used a microfluidic device (schematically shown in Supplementary Figure S2A) fabricated using Polydimethyl-siloxane (PDMS) by following conventional soft lithography techniques [52]. The device consisted of an array of microreservoirs, that were primed with tumor derived CM, establishing a chemokine gradient to prompt directional neutrophil migration through the connecting microchannels. A suspension of neutrophils at different cell densities (1 × 10^6^, 5 × 10^6^ and 1 × 10^7^ cells/mL) were tested for device characterization. However, for all functional comparative assays, the cell concentration of 1 × 10^7^ cells/mL was used. During the assay, the device was submerged in RPMI media and maintained in a humidified environment with 5% CO_2_ at 37 °C for the required duration. NETs formation within the device was visualized using 2.5 µM Sytox Orange (S11368, Invitrogen, Waltham, MA, USA) added to the CM. To selectively quantify the NETs produced by the non-migrated cells within the microchannels, fresh media was passed through the device using a sterile syringe and cells were collected from the outlet for immunostaining.

#### 2.2.2. Neutrophil-Tumor Spheroid Interaction Assay

To recapitulate the in vivo-like tumor-neutrophil interactional behavior, we used a modified Tumor-immune Microenvironment-on-Chip (TIME-on-Chip) [12]. Herein, active tumor spheroids were generated within a composite hydrogel-glass cover plate assembly and embedded within a collagen matrix. Spheroids of MDA-BrM and MDA-231 were created using the low attachment polyacrylamide (PAAm) microwell plates [12,53]. Tumor cell suspensions (at a concentration of 5 × 10^6^ mL^−1^) were seeded onto the PAAm microwell surface and the setup was left undisturbed for 5 min to allow the cells to settle into the microwells by gravity. Given that untreated PAAm resists protein adhesion, cells present within the non-adhesive microwells spontaneously aggregated to form spheroidal tumor clusters. Excess cells on the surface of the microwell plate were gently washed away using culture media, and the setup was stored inside the incubator for allowing the tumor spheroids to compact over 24 h in preparation for further assays. Thereafter, collagen was added on top of the spheroids, and the microwell plates were magnetically attached to a track-etched hydrophilic porous membrane set-up (having 3 µm pores) using appropriate O-ring spacers. Prior to the assays, freshly isolated and labelled neutrophils were added on top of the porous membrane and the devices were incubated at 37 °C, for further analysis at the different time points, as required.

### 2.3. Immunostaining

The microwell plates containing the spheroids were initially washed in PBS and fixed in 4% (*v/v*) paraformaldehyde in PBS at room temperature. Thereafter, the devices were washed twice with PBS for 5 min each and permeabilized with 0.1% (*v/v*) Triton-X in PBS for 1 h at room temperature. After washing 2 times with PBS for 5 min each again, the spheroids were blocked against non-specific binding with 2.5% (*v/v*) goat serum in PBS for 1 h at room temperature. Devices were then incubated overnight at 4 °C with rabbit anti-Histone H3 (Citrulline Arg17, Citrulline Arg2, Citrulline Arg8) antibody (NB10057135, Novus Biologicals, Littleton, CO, USA) in a 1:500 dilution of goat serum. The samples were incubated at room temperature for 2 h with goat anti-rabbit IgG (H + L) secondary antibody, FITC (11-4839-81, Invitrogen) in a 1:500 goat serum dilution. The samples were rinsed for 10 min in PBS.

### 2.4. Quantitative Reverse Transcription Polymerase Chain Reaction (qRT-PCR)

Total RNA was isolated from neutrophils using Aurum total RNA mini kit (Bio-Rad, Hercules, CA, USA). Reverse transcription was performed using iScript cDNA Synthesis kit (Bio-Rad #1708891). PCR reactions were performed in a CFX96 Real-Time PCR Detection System (Bio-Rad), using iTaq Universal SYBR Green Supermix, with the following primers: Actin: (5′-CACCATGGATGATGATATCG-3′ and 5′-GAATCCTTCTGACCCATGC-3′) and PrimePCR™ PreAmp for SYBR^®^ Green Assay: CXCR2, Human (Bio-Rad qHsaCED0022673). Three independent PCR reactions were performed for each transcript.

### 2.5. Cohort Analysis

For cancer cohort analysis, we generated a microarray dataset of 710 patients from Gene Expression Omnibus (accession numbers: GSE12276, GSE2034, GSE2603, GSE5327, and GSE14020). These datasets were all normalized using MAS5.0, and each microarray was centered to the median of all probes.

### 2.6. Imaging and Statistical Analysis

Both brightfield and fluorescent imaging were carried out using an inverted fluorescence microscope coupled with a CCD camera (Olympus IX-83, Tokyo, Japan). CellSens image analysis software (Olympus, Tokyo, Japan) was used for data acquisition. Image processing and analyses were completed using Image-J (NIH, Bethesda, MD, USA). In all experiments, neutrophils count was obtained by calculating the total number of Hoechst signaling cells. For all migration experiments, the percentage of neutrophil migration was estimated by taking the fraction of the number of migrated neutrophils to the estimated total neutrophils available within ~500 µm from the tumors. The total percentage of NETs occupation within a microscopic field of view (visualized upon immunostaining for Citrullinated H3 (H3Cit)). The experimental data for the neutrophil parameters (neutrophils count or % NETs) for different experimental conditions are expressed as mean ± standard error (SEM) unless otherwise specified. In general, comparative data analysis of populations was performed without pre-specifying a required effect size. Datasets were normally distributed, with similar variances between compared groups. All statistical analysis was conducted using student’s *t*-test or two tailed one-way ANOVA analyses with Tukey post-hoc pairwise comparisons (Prism; GraphPad Software, La Jolla, CA, USA). The *p*-values less than 0.05 were considered significant.

## 3. Results 

### 3.1. Comparison of Neutrophil Responses to Conditioned Media Derived from MDA-BrM and MDA-231

Neutrophil response to tumors is influenced by the autocrine and paracrine signaling within the TIME, and different tumor types and subtypes possess inherently distinct cytokine expression profiles that could drive distinct neutrophil behavior [23,24,54]. This prompted us to firstly investigate the neutrophil recruiting ability of MDA-BrM and MDA-231. For this assay, we studied chemotaxis response of neutrophils to the respective tumor conditioned media (TCM), using an established microfluidic chemotaxis device [52]. Herein, the directional chemotaxis was quantified by counting the total number of cells that migrate over 24 h into the microfluidic reservoirs filled with the respective TCMs (Schematically shown in Supplementary Figure S2A(i)).

**Figure 2:**
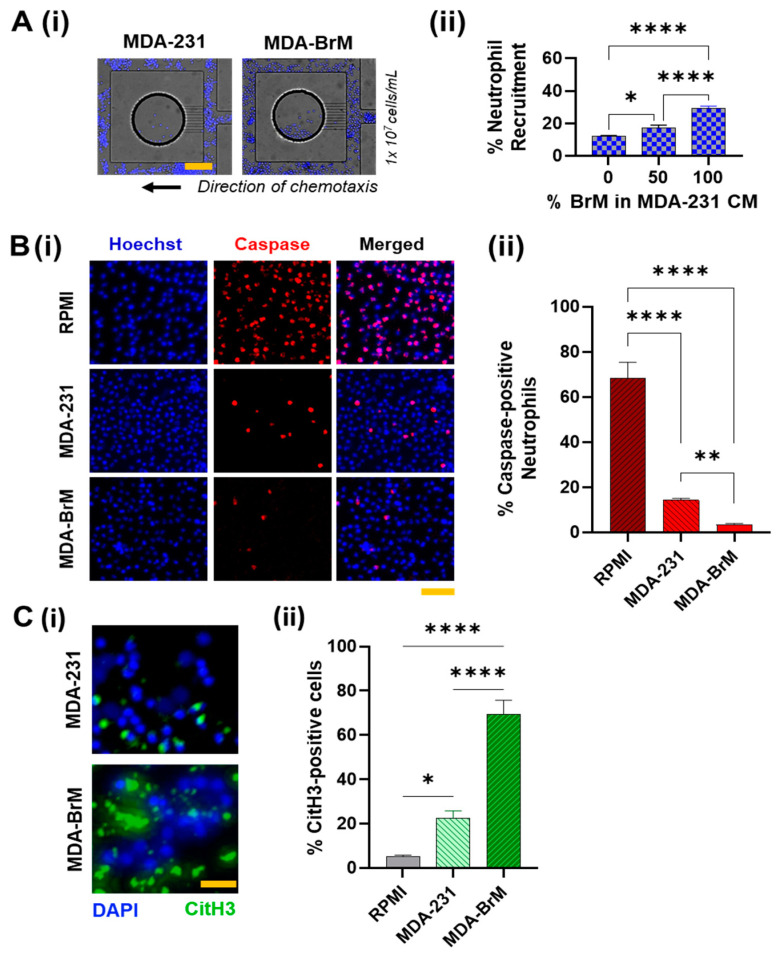
Experimental characterization of neutrophil responses to tumor derived conditioned media. (**A**) (i) Representative images of the microfluidic device showing the neutrophils migrated into the reservoirs (circular) containing the respective tumor-derived conditioned media (TCM), with an initial cell loading density of 1 × 10^7^/mL. Scale bar represents 100 μm. (ii) Quantification of neutrophil chemotaxis in terms of the average percentage of the cells that migrated into the microfluidic reservoirs containing respective TCM (whose composition varied by the percentage of conditioned media (CM) derived from MDA-BrM mixed with MDA-231-CM) from within 500 μm proximity. (**B**) (i) Representative images of neutrophils (stained Hoechst blue) incubated in the respective TCM, labelled for Caspase-3/7. Scale bar represents 50 μm (red) (ii) Significantly lower neutrophil population exhibiting Caspase/+ signaling in MDA-BrM-CM than MDA-231-CM (*p* < 0.01) or RPMI (*p* < 0.0001). (**C**) (i) Representative images of neutrophils (stained Hoechst blue) incubated in the respective TCM, immunostained for citrullinated histones H3 (citH3) (green). Scale bar represents 50 μm (ii) Significantly greater neutrophil population incubated in MDA-BrM-CM produced NETs, than MDA-231-CM or RPMI (*p* < 0.0001), as identified by the citH3 signaling. (All data collected from at least *n* = 3 repeats of *N* = 3 experiments, mean ± SEM. Significance was determined using one-way ANOVA with Tukey’s post-hoc analysis * *p* < 0.05, ** *p* < 0.01, **** *p* < 0.0001, ns = not significant).

Neutrophil chemotactic response toward both the TCMs was evident by the migration of the neutrophils into the CM reservoirs. (Figure 2A(i)). In addition, as expected, the population of neutrophils migrating toward both the CMs increased with the initial cell seeding density (Supplementary Figure S2A(ii)), suggesting that inter-cellular proximity [55] could regulate neutrophil chemotaxis. For a loading density of 1 × 10^7^ cells/mL, we observed that CM from MDA-BrM elicited almost twice (~32%) the neutrophil migration than MDA231-CM (~15%) (with *p* < 0.01). We tested the chemotaxis of neutrophils in three heterogeneous compositions of the TCM’s consisting of 0, 50, 100% of MDA-BrM -CM in MDA-231-CM. We observed that the percentage of neutrophil migration increased with an increase in the fraction of BrM in the CM cocktail, suggesting that the active factors secreted by MDA-BrM drive neutrophil infiltration into the tumor with greater potency than MDA-231 (Figure 2A(ii)).

However, for all conditions, we found not all neutrophils in the proximity of reservoirs were being recruited. We speculated that neutrophils exposed to the TCM’s could undergo spontaneous cell death [56,57] in which case, they could be rendered non-viable to undergo chemotaxis. Caspase-3 activation during neutrophil death is well documented [58]. Caspase-3/7 activation is responsible for the initiation of the degradation phase of apoptosis [59], and can be used as a specific marker for identifying pro-apoptotic cells. Therefore, to quantify the neutrophils’ health when exposed to the TCMs, we incubated the neutrophils in the respective TCMs for 24 h, and thereafter, we stained the neutrophils for Caspase-3/7 [60]. Primary neutrophils in culture showed the most caspase/+ signal, as expected. In comparison, cells incubated in TCMs showed lesser caspase positive signals, possibly due to the pro-inflammatory mediators in the TCM retarding spontaneous neutrophil death. However, the number of neutrophils with caspase/+ signaling in MDA-BrM was significantly lower (almost negligible) than MDA-231. Taken together with the chemotaxis results presented earlier, we inferred that TCM derived factors from MDA-BrM support more favorable anti-cytocidal neutrophil behavior, possibly for eliciting greater neutrophil infiltration into the tumor.

To further corroborate our observation, we studied the apoptotic behavior of the neutrophils by staining the cells with Annexin-V after 24 h TCM treatment. Contrary to the previous results, we observed more Annexin-V/+ neutrophil population in the TCM from MDA-BrM (~70%) than MDA-231 (~40%) (Supplementary Figure S2B). Intriguingly, we also observed that in MDA-BrM, most Annexin-V/+ neutrophils appeared to be enlarged, with a swollen morphology (quantified in Supplementary Figure S2C). We hypothesized that the neutrophils cell membrane could be disrupted, enabling Annexin-V binding to the phosphatidylserine (PS) from the cytoplasmic side of the plasma membrane [61]. This prompted a speculation that neutrophils could possibly undergo NETosis [62]. Therefore, to compare the NETosing potential of the neutrophils in the respective TCMs, we immunostained the cells for citrullinated Histone H3 (citH3), a standard NETs marker. The results showed that, compared to MDA-231 (~21%), a significantly greater (*p* < 0.001) percentage of neutrophils underwent NETosis upon culture in TCM derived from MDA-BrM (~70%), with a significantly larger average spread area (Supplement Figure S3A). To extrapolate this static culture behavior to dynamic conditions, we stained the neutrophils in our microfluidic chemotaxis assays to observe if they underwent NETosis inside the channel. Thereafter, we flushed the microfluidic channel with culture media to collect the non-migrated cells present in the main channel and collected the migrated cells from inside the TCM reservoirs by carefully detaching the device. Upon immunostaining the neutrophils for CitH3, we observed similar behavior: the neutrophils from MDA-BrM device showed significantly greater NETs signaling than MDA-231 (Supplementary Figure S3B).

Taken together, our results suggest that BrM could retard spontaneous neutrophil apoptosis and induce greater neutrophil chemotactic infiltration into the tumor niche and activate the formation of NETs.

### 3.2. Involvement of CXCR2 in BrM-Neutrophils Interactions

To identify the potential tumor-derived factors that could be driving the differential neutrophil responses, we evaluated the differences in the cytokine expressions between the parental tumor and the brain metastatic subtype. Previous works have shown that some CXC ligands were present at higher levels in more aggressive forms of human breast cancers [63,64,65,66]. Cancer cells that overexpress CXCL1 and 2 are primed for survival in metastatic sites [35]. Therefore, we decided to evaluate the expression profiles of the CXC ligands in the parental tumor and the brain metastatic subtype. For this study, we generated a microarray dataset of 710 patients from the Gene Expression Omnibus (GEO) database, and mRNA expression of CXCR2 ligands were examined in breast cancer patients with (*n* = 47) or without brain metastasis (*n* = 315).

**Figure 3:**
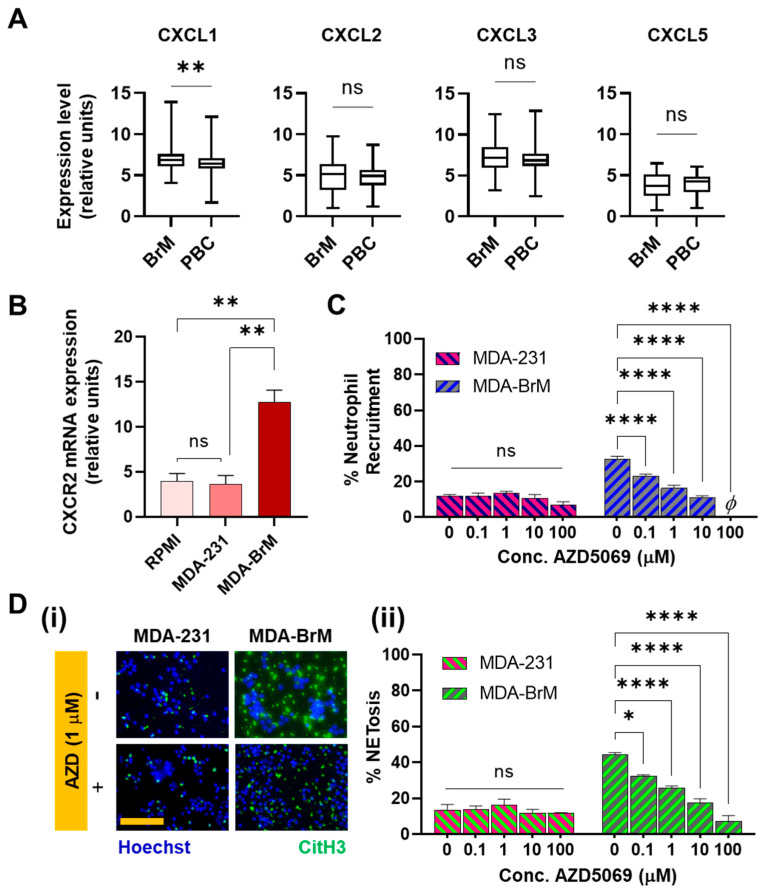
(**A**) mRNA expression of CXCR2 ligands were examined in breast cancer patients with (*n* = 47) or without brain metastasis (*n* = 315). Significantly higher expression (*p* = 0.0058) of CXCL1 was observed in brain metastasis compared to parental breast cancers. (**B**) mRNA expression of CXCR2 was measured in human neutrophils treated with indicated conditioned media. Incubation in MDA-BrM-CM resulted in significantly greater expression of CXCR2 in neutrophils than MDA-231 and the regular RPMI culture media. (**C**) Upon treatment of neutrophils with AZD5069, neutrophil recruitment to MDA-BrM-CM reduced significantly in a dose dependent manner, whereas no significant difference was observed in neutrophil recruitment to MDA-231-CM (**D**) (i) Representative images of human neutrophils cultured in tumor conditioned media doped with AZD5069, showing the neutrophils (Hoechst blue) and NETs (citH3 Green). Scale bar represents 100 μm. (ii) No significant differences were observed in the NETs production by neutrophils incubated in AZD5069-doped MDA-231-CM, whereas, with increasing concentration of AZD5069 doping the NETs density reduced significantly in neutrophils cultured in MDA-BrM conditioned media (Data collected from at least *N* = 3 experiments, mean ± SEM. Significance was determined using one-way ANOVA with Tukey’s post-hoc analysis * *p* < 0.05, ** *p* < 0.01, **** *p* < 0.0001, ns = not significant).

The data showed that brain metastatic breast cancers expressed significantly increased (*p* = 0.0058) levels of CXCL1 (Figure 3A). This finding led us to hypothesize that neutrophil functionalities in brain metastatic tumors could be significantly regulated by the corresponding receptor CXCR2 [67], which is the main chemokine receptor on neutrophils for CXCL1 [68]. For other CXCR2 ligands, such as CXCL3 and CXCL5, RNA levels were not significantly different in brain metastatic tumors compared to the parental, though Xing et al. [51] reported the upregulation of Interleukin-8 levels in BrM together with CXCL1. Therefore, to evaluate the CXCR2 expression, neutrophils were incubated in respective tumor conditioned media (TCM) and qRT-PCR was performed. As judged by the results (Figure 3B), a significantly more elevated expression (*p* < 0.01) of CXCR2 was found in the neutrophils incubated in BrM than in PBC.

This confirmed the involvement of the CXCR2 axis in the interaction of neutrophils with BrM and led us to further investigate the effects of inhibiting CXCR2 on the BrM-specific neutrophil responses. We therefore repeated the chemotaxis (microfluidic) and the NETosis assays, upon treating the neutrophils with AZD5069 [69], a selective small-molecule antagonist of the CXCR2 chemokine receptors. The results showed that the blockade of CXCR2 receptors using AZD5069 profoundly inhibited recruitment of neutrophils to the BrM conditioned media in a dose dependent manner (Figure 3C). However, to MDA-231, AZD5069 produced negligible changes in the neutrophil chemotaxis, corroborating the observations made in SenGupta et al. (2021) [54]. Furthermore, when neutrophils treated with AZD5069 were incubated in the respective TCM’s for 24 h, we also observed that the density of CitH3+ features in BrM-CM decreased upon treatment with AZD5069 in a dose dependent manner (Figure 3D). We observed significant attenuation of chemotaxis (*p* < 0.0001) and NETosis (*p* < 0.05) responses to BrM even with 0.1 µM used for treatment. In contrast, no significant changes were observed in the manner of NETs formation, for AZD5069-treated neutrophils incubated in MDA-231 CM (Figure 3D(ii)). 

Therefore, taken together, our data show a strong involvement of the CXCR2 axis in modulating neutrophil chemotaxis and NETosis responses specific to brain metastatic variants, which can be attributed to the increased expression of CXCR2 ligands in the BrM compared to the parental tumors.

### 3.3. Distinct Spatio-Temporal Dynamics of Tumor-Associated Neutrophils in BrM

**Figure 4:**
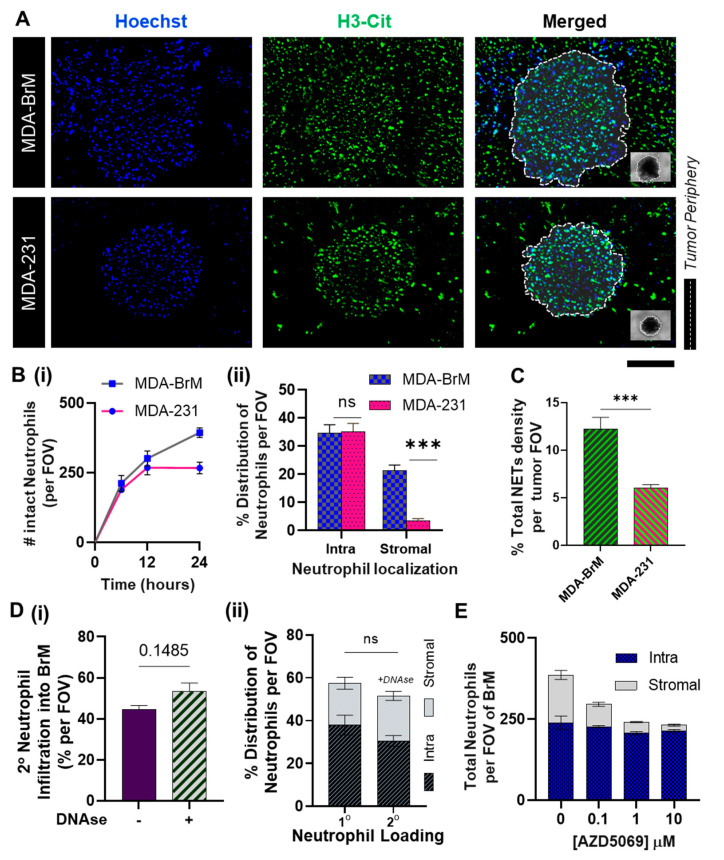
Spatio-temporal dynamics of the neutrophil response to BrM demonstrated using the Tumor-Immune Microenvironment-on-Chip (TIME-on-Chip). (**A**) Representative images of the tumor spheroids showing the infiltration of neutrophils (stained Hoechst blue) and the NETs generated (immunostained for citH3 green) within the tumor niche. Scale bar represents 100 μm. (**B**) (i) A count of the total intact neutrophils that infiltrated into the respective tumors, quantified within the field of view, within the first 12 h, no significant difference was observed in the neutrophils that migrated toward the tumors, after 24 h, a significantly greater neutrophil population was observed in MDA-BrM, than MDA-231. (ii) At the 24 h time point, among the infiltrated neutrophils, no significant difference was observed in the total number of tumor-contacted (intraspheroidal) neutrophils, whereas accumulation of neutrophils in the stromal region was significantly greater for MDA-BrM than MDA-231. (**C**) The total percentage FOV covered by NETs was significantly greater (*p* < 0.005) for MDA-BrM than MDA-231 (for at least *n* = 9 spheroids measured over three experiments, mean ± SEM, *t* test). (**D**) NETs formed around the BrM spheroids were digested using DNAse, and a 2° round of neutrophils were introduced to observe their tumor-infiltrating behavior (i) No significant difference was observed in the total neutrophil infiltration with or without DNAse treatment (ii) the spatial distribution (i.e., stromal and intra-spheroidal distribution) of 2° neutrophil accumulation in the BrM tumor region was similar to the first round of neutrophil loading. (**E**) Treatment with AZD5069 reduced the total neutrophil infiltration into the BrM; however, with AZD5069 treatment, significant reduction (*p* < 0.01) was observed in the neutrophil localization within the stroma and not the intra-spheroidal neutrophil accumulation. (Total *n* = 9 spheroids measured over 3 experiments, mean ± SEM, *t* test) (Data collected for *n* = 9 spheroids from *N* = 3 experiments, mean ± SEM. Significance was determined using one-way ANOVA with Tukey’s post-hoc analysis *** *p* < 0.001, ns = not significant).

While the results thus far focused on the individual modalities of tumor-related neutrophil functionalities, we sought to simulate the effects of CXCR2 activation on the wholesome neutrophil behavior within a breast tumor microenvironment consisting of brain-tropic metastatic variants. For this study, we conducted in vivo-like three-dimensional neutrophil-tumor interactional assays using a modified Tumor-Immune Microenvironment-on-Chip (TIME-on-Chip) [12]. Herein, (as schematically shown in cross-section in Supplementary Figure S4A), spheroids of MDA-BrM and MDA-231 were created separately within hydrogel microwell platforms and embedded within collagen matrices. In each device, neutrophils were seeded onto a microfluidic channel constructed on a porous membrane that was integrated on top of the collagen matrix. The neutrophils’ response to the respective tumor spheroids over 24 h was quantified by calculating the total number of neutrophils that migrated across the membrane and infiltrated the tumor through the ‘stromal’ collagen layer. As seen in Figure 4A, neutrophil infiltration into the tumor region and the production of NETs were observed for both MDA-BrM and MDA-231. The migrated neutrophils created a distinct spatial distribution within the tumor region, as they either localized onto the densely packed tumor spheroids (addressed to as ‘intratumoral’ or ‘intra’) or populated the peripheral collagen region surrounding the spheroids (‘stromal’) within a maximum distance of 250 μm from the tumor margin (field of view).

To study the temporal migratory dynamics, we counted the total intact neutrophils present within the tumor region at different time points (Figure 4B(i)). Within the first 12 h of the co-culture, no significant difference was observed in the total neutrophil infiltration between the tumor types. However, at the end of 24 h, 55~60% of the neutrophil population were present intact on and around BrM spheroids, which was significantly greater (*p* < 0.005) than the total neutrophil infiltration observed with MDA-231 spheroids (35~40%). A large population of the neutrophils accumulated in the peripheral collagen region surrounding the spheroids (Figure 4B(ii)), which ultimately established the difference in the overall count of intact neutrophils that infiltrated into the respective tumors. No significant differences were observed in tumor-contacted neutrophils count between the tumor types (Figure 4B(ii)). 

At the same time, we observed that the overall NETs density, (i.e., % FOV covered by citH3) was significantly higher (*p* < 0.001) in MDA-BrM than MDA-231 (Figure 4C), as expected based on the results of our TCM static culture assays. We speculated that the increased NETs formation on and around the MDA-BrM spheroids could hinder [46] further neutrophil recruitment onto the spheroids, thus causing the neutrophils to accumulate in the stromal region. To rule out this possibility, we conducted a two-step sequential assay series wherein we firstly co-cultured the neutrophils with the BrM spheroids over 24 h, treated the setup with NETs digesting DNAse, and thereafter integrated the setup with the porous membrane seeded with a second round of neutrophils (2°). To distinguish between the two neutrophil populations, only the 2° neutrophils were stained blue (Hoechst). DNAse treatment did not significantly alter (*p* = 0.1485) the neutrophil recruitment from the second loading (Figure 4D(i)). Approximately 50~55% of neutrophils from the second loading were recruited into the tumor region. In addition, no significant difference was observed (*p* = 0.4302) in terms of the neutrophils’ infiltration with the first loading, and moreover the neutrophils also displayed a similar behavior with their intra-tumoral and stromal spatial localization. This suggests that the formation of NETs did not limit the neutrophils from contacting the tumor, leading to speculations that the spatial distribution of the neutrophils in MDA-BrM is possibly tumor-mediated. 

Against this background, we investigated how a CXCR2 blockade would affect the spatial accumulation patterns [70] of the neutrophils in the BrM model. Therefore, in our next co-culture assay, we treated the neutrophils with AZD5069 prior to loading. The results (Figure 4E) showed that AZD5069 treatment significantly decreased the stromal neutrophil count in the BrM, with no significant decrease in the tumor-contacted neutrophils (Supplementary Figure S5). Thus, the expected overall reduction in the neutrophil infiltration was coming from the reduction of the stromal neutrophils, and within the chosen concentration range of AZD5069. Interestingly, we found no difference in the number of intratumoral neutrophils in the BrM. 

Therefore, from our results, we postulate that within a heterogeneous tumor, the brain-tropic breast tumor variants could polarize a distinct subset of a homogeneous neutrophil population into a Tumor-Associated Neutrophil (TAN) phenotype that could exhibit distinct spatio-temporal dynamics based on the levels of CXCR2 activation.

### 3.4. Inhibition of NETs in BrM Limits Tumor-Associated Neutrophil Infiltration

The overall understanding of the extent to which the presence of TANs within the TIME could mediate tumor aggressiveness and progression is currently evolving [23]. However, metastatic breast cancer cells are known to induce metastasis supporting NETs [45] and it has also been reported that CXCR2 agonists within TIME are the major mediators of tumor-induced NETs. Our data (Figure 4C) shows that small molecule CXCR2 antagonist AZD5069 not only effected a significant decrease in the influx of TANs toward the BrM tumor, but also a reduction in the overall density of the NETs produced by the infiltrating neutrophils. In line with these findings, we therefore sought to further understand if AZD5069 treatment of the neutrophils had a complementary effect on the BrM tumor response. A 3D collagen matrix migration assay is a versatile method to analyze the migration of cells within a physiological-like 3D environment [71,72]. Therefore, using our TIME-on-Chip platform, we observed the tumor response by measuring the migration and invasion of the BrM spheroids into the collagen region over 24 h. For quantification, we used the spheroid distortion parameter (ϕ), defined as the change in the projected area of the BrM spheroids after migration, over the initial spheroid area, as viewed under the microscope [12].

**Figure 5:**
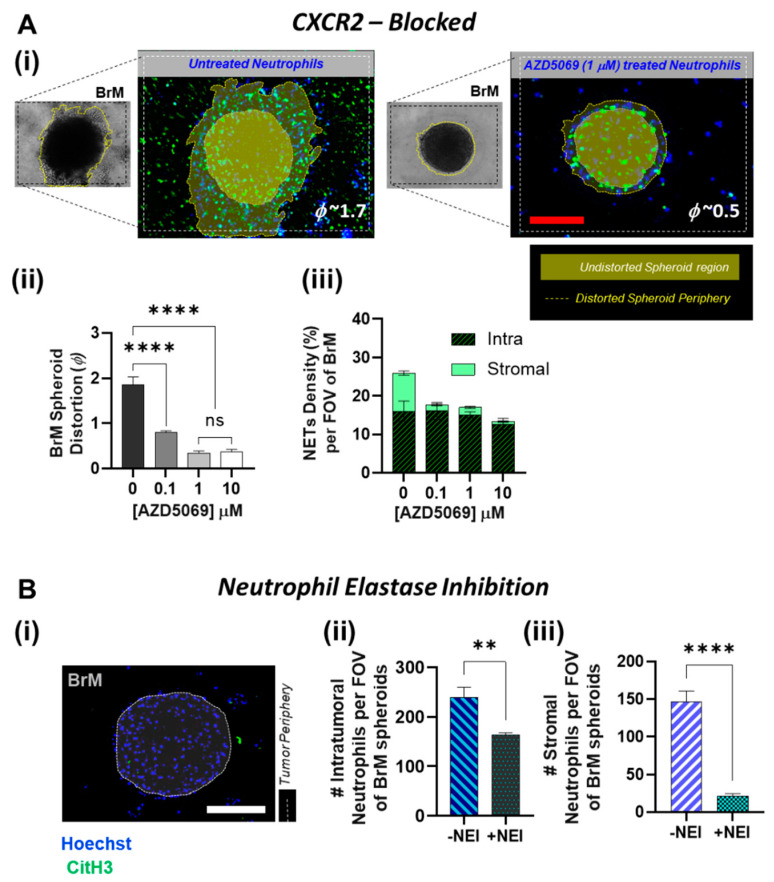
CXCR2-mediated NETs formation and the reciprocal BrM tumor response. (**A**) (i) Representative images of the spheroids (brightfield), showing the infiltration of neutrophils (stained Hoechst blue) and the NETs generated around the spheroids (labeled with citH3 green). Invasive domains (the distorted spheroid periphery) are marked by the dotted lines, and the undistorted spheroid contour prior to tumor invasion is highlighted in the fluorescent images. The invasive domain is distinct by its presence within the stromal collagen region seen out of plane from the rest of the spheroid. The BrM response is measured using the spheroid distortion parameter (ϕ). Treatment of neutrophils with AZD5069 reduces BrM migration and invasion, as qualitatively shown. (ii) Quantification of variation in BrM spheroid distortion upon treating the neutrophils with AZD5069 (iii) the overall % of the tumor region covered by NETs was significantly reduced (*p* < 0.001) when neutrophils were pretreated with AZD5069. For the chosen drug concentration range, no significant difference was observed in the NETs density for the intra-tumoral NETs, whereas the formation of stromal NETs was abrogated significantly; almost negligible stromal NETs was observed even for AZD5069 concentration of 0.1 μM (for total *n* = 9 spheroids measured over 3 experiments, mean ± SEM, *t* test). (**B**) (i) BrM spheroid distortion is significantly reduced (*p* < 0.0001) by abrogation of NETs with NEI pre-treatment of the neutrophils. (ii), (iii) A comparison of the NEI-treated neutrophil infiltration and localization within the FOV of the BrM tumor region shows NEI treatment significantly reduced the neutrophil infiltration into both the intra-tumoral and the stromal regions of the intact spheroids (for total *n* = 9 spheroids measured over 3 experiments, mean ± SEM, *t* test). (Scale bars represent 100 μm. Data collected for *n* = 9 spheroids from *N* = 3 experiments, mean ± SEM. Significance was determined using one-way ANOVA with Tukey’s post-hoc analysis ** *p* < 0.01, **** *p* < 0.0001, ns = not significant).

In the absence of any infiltrating neutrophils, BrM showed negligible distortion, and as expected in the presence of NETs, BrM cells migrated into the collagen regions shown in Figure 5A(i). However, upon treatment of neutrophils with AZD5069, we observed a significant decrease (*p* < 0.0001) in the spheroid distortion. Even with 0.1 µM AZD5069 treatment, the distortion parameter dropped significantly (from 1.7~2 to 0.7~0.9), and almost negligible spheroid distortion was observed when a concentration greater than 1 µM AZD5069 was used (Figure 5A(ii)). Therefore, clearly, blocking CXCR2 in neutrophils has a significant effect on the BrM tumor behavior. However, this data was not confirmatory to delineate the cause of attenuation in the tumor invasion as being a result of AZD5069-targeted reduced NETosing capability of the TANs, or being due to the overall decreased availability of the neutrophils/NETs around the spheroids in general.

Therefore, in our next tumor response assay, we controlled the baseline spatial distribution by adding the AZD5069-treated neutrophils directly into the collagen, thereby ensuring a uniform initial distribution of neutrophils around the spheroids. As expected, tumor migration was dependent on the NETs formation, and the reduction in the overall NETs density with AZD5069 treatment correlated with the diminished spheroid distortions. The number of spheroid-contacted neutrophils also decreased with AZD5069 treatment. However, interestingly, the NETs density (% area covered by NETs) distribution data (Figure 5A(iii)) showed that, whereas no significant difference existed in the tumor-contacted NETs density between untreated and AZD5069 treated neutrophils within our concentration range (0–10 µM), the BrM-induced stromal NETs diminished significantly (*p* < 0.001) with an increase in AZD5069 concentration. With untreated neutrophils, BrM induced NETs-formation covered almost ~10% of the surrounding stromal region. However, even with 0.1 µM AZD5069 treatment, negligible NETs signaling from the stromal region was observed. 

In our previous work, we had demonstrated that the availability of stromal NETs and not the tumor-contacted NETs, is critical for inducing tumor invasion [12]. Similarly, we predicted that CXCR2-mediated accumulation of the TANs in the stroma could be a pro-NETotic mechanism of the BrM to initiate tumor invasion. To further probe this behavior, we abrogated NETs formation in the neutrophils by treating them with 10 µM Sivelestat [73,74], a Neutrophil Elastase Inhibitor (NEI), and we observed the neutrophil infiltration and their spatial localization behavior in response to the BrM spheroids. As shown in Figure 5B, NEI treatment significantly decreased the influx of the neutrophils into the BrM for both tumor-contacted (*p* < 0.01) and the stromal neutrophils (*p* < 0.001). Negligible TAN accumulation was observed in the BrM stroma. On the contrary, the number of neutrophils infiltrating the MDA-231 showed no significant difference in both stromal and intraspheroidal distribution, with or without NEI treatment (Supplementary Figure S6). Therefore, the CXCR2-mediated infiltration and the formation of tumor-aiding NETs by the TANs in a location-dependent manner can be reversed by inhibition of NETs.

## 4. Discussion 

There is an increasing recognition of the diversity in neutrophils’ functionalities and their plasticity within the pathology of tumors. Emerging evidence indicates that different subpopulations of neutrophils such as low-density neutrophils [75] or the polymorphonuclear-myeloid-derived suppressor cells resembling the neutrophils [76] are actively involved in cancer growth and metastasis [75,76,77,78]. Given that the plasticity of neutrophils enables them to adapt to different cancer microenvironments and exert different effects on cancer development, delineating the neutrophil heterogeneity and its interplay within the TIME could enable the discovery of new mechanisms of metastasis and help develop suitable immunotherapeutics by targeting neutrophils and their specific subtypes [77,78,79,80]. The purpose of this study was to demonstrate how the mechanistic functionalities of a homogeneous population of neutrophils could dynamically acquire heterogeneity within a TIME, based on the metastatic potential of the tumor cells.

Tumor-Associated Neutrophils (TANs) have been detected in varying degrees within the tumor niche of various kinds of malignancies, including breast cancers [81,82,83]. Yet, cancer-associated biomolecular cues that drive neutrophil functionalities within a primary breast tumor niche are not well defined. Comprehensive understanding of the intrinsic neutrophil responses to specific pro-metastatic variants of the parental breast tumors could possibly shed light on potential strategies for the better management of neutrophil inflammation and regulation of breast cancer metastasis. In this paper, we have shown that CXCR2 in neutrophils is upregulated in response to the overexpression of the corresponding ligands in brain metastatic variants of breast cancers. We have demonstrated the mechanisms by which CXCR2 signaling could modulate the spatio-temporal dynamics of the neutrophil responses toward brain-tropic breast cancers. These observations are also in line with some of the recent findings available in the literature that support CXCR2 antagonism as an approach to regulate the anti-tumoral TAN behavior [54,70,84,85]. Furthermore, treatment with different CXCR2 ligands has been shown to enhance NETs formation [46], which is another pro-tumoral/pro-metastatic activity of neutrophils. CXCR2 stimulation induces nuclear translocation of neutrophil elastase, and enhanced citrullination of histones that triggers chromatin decondensation during NET formation [86]. Here, we show that inhibiting NETs formation through Neutrophil Elastase Inhibitor negates the effect of CXCR2 activation in the neutrophils. Therefore, based on our results, we speculate that CXCR2 activation could be used by the metastatic tumors as a mechanism to retard apoptosis and program the tumor-infiltrating TANs into a pro-NETotic state, to assume a unique spatial distribution that assists in the subsequent migration and invasion of the metastatic tumor cells. A similar observation has also been made by Sody et al. [70]; blocking CXCR2 using AZD5069 profoundly inhibited recruitment of Tumor-Associated Neutrophils (TANs) into peripheral region surrounding solid tumor cell lesions within a mouse model, while intratumoral infiltration was only transiently attenuated and rebounded at later time points. Thus, our results demonstrate the existence of a complex tissue-level interplay between metastatic tumor development and the spatio-temporal dynamics of TANs and NETs, which represents an interesting problem warranting further studies. 

CXCR2 is a prominent chemokine receptor on neutrophils [87]. CXCR2 ligands play a multifaceted role within a TIME, and activation of CXCR2 and increased neutrophil migration has been associated with breast cancer metastasis [88]. Apart from CXCL1, discussed in this paper, CXCL8 or IL-8, which is also upregulated in BrM [51], is considered as one of the most potent neutrophil chemoattractants in inflammation that binds to CXCR2 [89,90]. Current research works are increasingly focusing on CXCR2 antagonism in therapeutic strategies for cancer and related diseases [91]. Interference with CXCR2/CXCR2-ligand interaction has been proposed as a means to limit the pro-tumorigenic activity of TANs [92]. Consistent with this idea, we found that pharmacological blockade of CXCR2 using small molecule antagonist AZD5069 significantly decreased the TANs influx towards the BrM, modulating their spatial localization within the intratumoral and stromal regions, and suppressing the metastatic invasion and dissemination. Even though CXCR2 is essential for neutrophil egression from the bone marrow and trafficking toward sites of inflammation [93,94], CXCR2 antagonism would not adversely affect the mobilization of neutrophils into the peripheral circulation, phagocytosis or the oxidative bursts [95]. Therefore, our data supports the potential of CXCR2 antagonists as a targeted treatment option for diseases in which neutrophils play a pathological role.

Specific to brain metastasis, treatment options of brain metastasis in breast cancers usually involve local modalities like surgical excision or radiation therapy (either stereotactic radiosurgery or whole brain radiation therapy). Systemic therapies have had a limited role in treatment due to the inability of several therapies to penetrate the blood brain barrier [96]. In recent times, several systemic agents have shown promising clinical activity either directly or indirectly against brain metastases in Her-2 positive breast cancers [97,98,99,100,101]. However, treatment options in triple negative disease are very limited and include capecitabine, eribulin, and bevacizumab with carboplatin or paclitaxel [102]. AZD5069, a selective and reversible CXCR2 antagonist, has been evaluated in respiratory conditions such as bronchiectasis, asthma, and COPD in differing doses. It was found to be well tolerated in all these early phase II trials [103,104,105]. In cancers, Durvalumab (MEDI4736), an anti-PD L1 (programmed death ligand 1) monoclonal antibody was evaluated in combination with AZD5069 in patients with metastatic head and neck cancer and advanced solid tumors (NCT02499328). An ongoing study (NCT03177187) is evaluating AZD5069 in combination with Enzalutamide, an androgen receptor antagonist, in metastatic castration resistant prostate cancer. Findings from our experiments showed an inhibition of neutrophil recruitment to brain metastatic variants of the parental breast cancers, and likely the decreased tumor-promoting NETosing potential of neutrophils upon treatment with AZD5069. This suggests that AZD5069 could also be effective to treat brain metastasis, which could be driven by the immunosuppressive neutrophil activities at the metastatic site [49,106]. However, further studies are warranted to investigate the potential of AZD5069 to cross the blood brain barrier, and subsequently evaluate its applicability in treating brain metastatic lesions secondary to breast and possibly other solid malignancies.

Nevertheless, the inter-tumoral heterogeneity of cancers [107,108,109], and particularly brain metastasis of breast cancers, could manifest through vastly different modalities of metastasis formation, therapy resistance and clinical outcomes to treatments [8,102,110]. Since NETosis can also occur as vital NETs [111] that does not necessarily lead to neutrophil death, it is also possible that different neutrophil subtypes could show distinct metastasis-aiding NETs dynamics [112]. Consequently, a generic extrapolation of our current findings into all the brain metastatic breast tumor models and their systemic neutrophil-targeted therapeutic approaches may not be valid. However, we conducted additional functional assays using the brain metastatic variants of HER-2+ breast cancer cell line SKBR-3. As reported by Xing et al. [51], SKBR-3 Brain Metastatic (SKBR-BrM) subtypes significantly overexpressed CXCL1 in comparison to their parental versions. Further, the results of our 3D neutrophil chemotaxis and NETosis experiment using the TIME-on-Chip, (presented in Supplementary Figure S7) showed that spheroids of SKBR-BrM recruited significantly more neutrophils into the tumor than SKBR-3, with a substantial neutrophil accumulation in the stromal region. The overall NETs density per FOV was also significantly higher (*p* < 0.01) in SKBR-BrM compared to the parental SKBR-3. All these results are in line with our data generated using the MDA-MB231 cell line, motivating further dedicated in vitro research using other tumor cell lines and animal models, for understanding the consummate translational relevance of the findings from our present work.

Lastly, our work emphasizes the advantages of studying tumors using biomimetic cell culture platforms that offer unique insights into tumor behavior over the traditional assays. In general, using conventional drug screening approaches to develop effective anti-tumor therapeutics against the intricate degrees of tumor heterogeneity has been extremely challenging, resulting in a poor rate of translational successes from benchtop to clinical trials. The microfluidics-enabled devices [113,114,115] such as the TIME-on-Chip used in our work, are effective surrogates to simulate the disease behavior at different stages. These microscale culture platforms require small sample volumes and provide an in vivo-like microenvironment for conducting complex ex-vivo biomimetic assays with increased analysis throughput to, at the very least, screen out drug candidates that would eventually fail during the human clinical trials, thereby eliminating the need to circumvent ethical issues associated with the controversial animal studies. In the present format, our devices do not capture the study of extravasation process that include interactions between neutrophils and the vasculature/endothelial cells, which could also determine the spatial distribution of neutrophils in vivo. Furthermore, our simplified models also lack some of the cellular components of the tumor microenvironment (such as, Tumor Associated Macrophages, Cancer Associated Fibroblasts, etc.) that may interfere with tumor cell signaling [116,117]. However, with simple modifications and appropriate controls, these biomimetic devices enable sophisticated recapitulation of certain critical in vivo biological events in a physiologically relevant manner, and can be easily adapted into high-throughput drug screening formats.

## 5. Conclusions

In this work, we suggested the reasonable hypothesis that neutrophils could be reprogrammed into a metastasis-promoting state within a tumor microenvironment. Our results identify CXCR2 activation as a major regulator of not only the recruitment of Tumor-Associated Neutrophils toward brain metastatic variants of breast cancers, but also their propensity of NETosis with unique tumor-aiding spatio-temporal dynamics. Thus, CXCR2 is a critical target for suppressing neutrophilic inflammation in BrM. We also demonstrate the potential utility of the CXCR2 inhibitor in limiting the neutrophil responses to BrM, and the resultant management of tumors. This new perspective indicates that neutrophil reprogramming in the course of cancer treatment is a problem worthy of attention. Preventing or reversing the reprogramming of neutrophils may be a potential strategy for better management of brain metastatic breast cancers.

## Data Availability

The data presented in this study are available on request from the corresponding author.

## Supplementary Materials

The following supporting information can be downloaded at: https://www.mdpi.com/article/10.3390/cancers14030515/s1, Figure S1: NETs formation in brain metastasis of breast cancers in mouse model, Figures S2 and S3: Characterization of neutrophil responses to tumor conditioned media derived from parental and brain metastatic MDA-MB-231, Figure S4: Spatio-temporal neutrophil infiltration dynamics into the brain metastatic and parental MDA-MB-231 spheroids, Figure S5: Neutrophil response to brain metastatic MDA-MB-231 with AZD5069 treatment, Figure S6: Neutrophil response, upon treatment with Sivelestat, to parental and brain metastatic MDA-MB-231 spheroids, Figure S7: Neutrophil response to brain metastatic and parental HER2+ SKBR-3 spheroids.
