# Supplementary material for: CXCR2 Mediates Distinct Neutrophil Behavior in Brain Metastatic Breast Tumor
Source: Cancers (Basel). 2022 Jan 20;14(3):515. doi: 10.3390/cancers14030515 (PMC8833752; doi:10.3390/cancers14030515)
Supplement: Supplementary file 1 [file cancers-14-00515-s001.zip › cancers-1545482-supplementary.pdf]

cancers MDPI

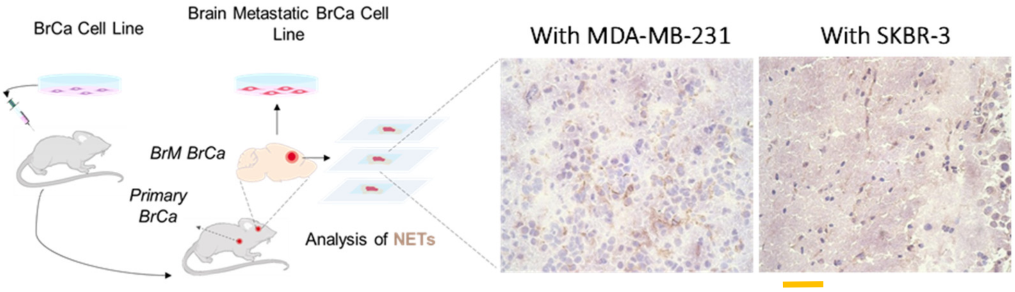

Figure S1: To firstly establish that neutrophils respond to brain metastatic breast cancers, Nude mice were inoculated with breast cancer cells, the triple negative MDA-MB231 and the HER2+ SKBR-3 cells through intracardiac injection. Upon sacrificing the mice on day 30, the brain was removed and sectioned by cryostat followed by staining for citrullinated histones H3 citH3. From the qualitative results, we not only observed the accumulation of neutrophils within the brain tissues, but the citH3 stains suggest that the neutrophils surrounding the brain metastatic breast tumors could produce NETs. Scale bar represents 20 μm.

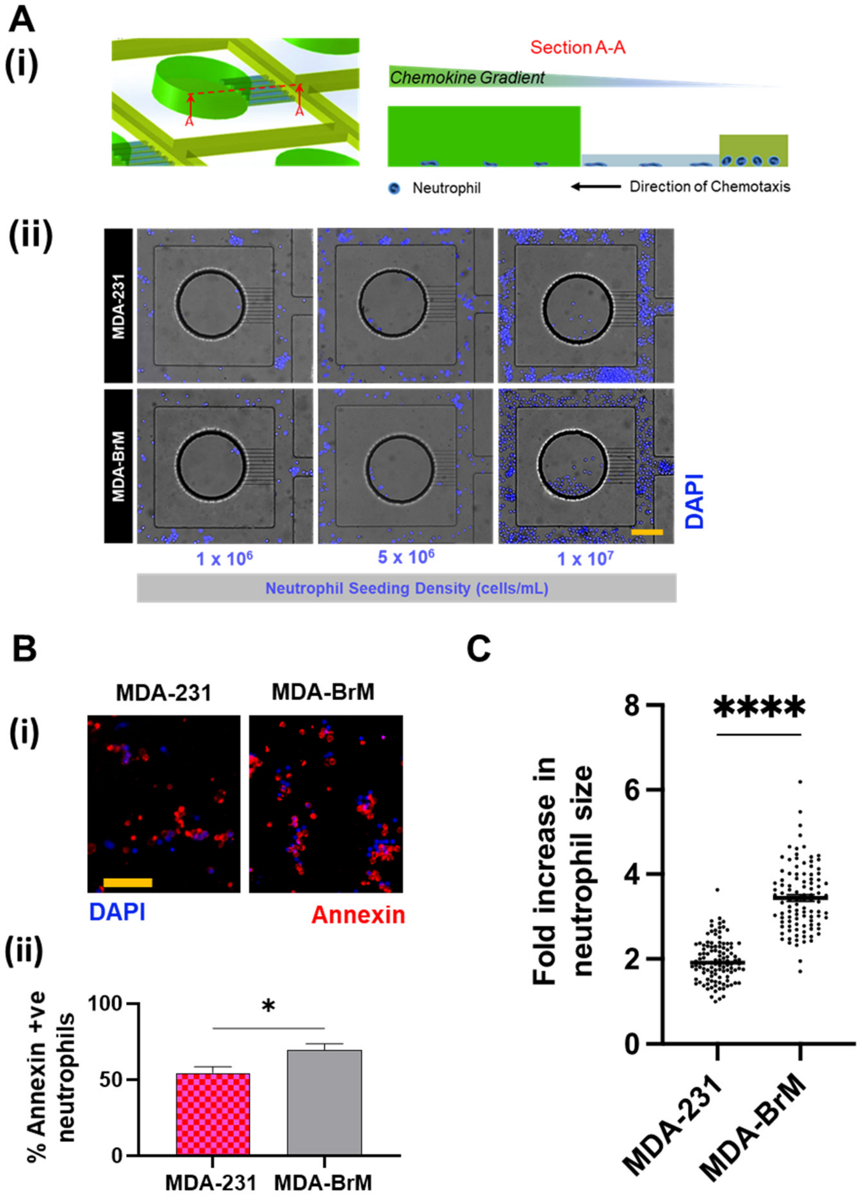

Figure S2: A (i) Schematic of the microfluidic device used in directional chemotaxis assays, consisting of a reservoir filled with tumor-derived conditioned media, and neutrophils in the main flow channel sense the chemokine and migrate toward the reservoir through the connecting channels. Section A-A shows the cross-sectional view and the schematic formation of chemokine gradient that drives the neutrophil chemotaxis. (ii) Representative images of the microfluidic device showing neutrophil chemotaxis toward the TCM for different cell loading densities. Scale bar represents 100 µm. **B.** (i) Representative images of neutrophils (stained blue) incubated in the tumor conditioned media and stained for Annexin –V (red). Scale bar represents 50 µm (ii) Cells in MDA-BrM derived TCM showed significantly greater ($p < 0.05$) Annexin/+ signaling and **C**. also appeared significantly more swollen ($p < 0.001$) than MDA-231. (All data collected from at least $n = 3$ repeats of N = 3 experiments, mean ± SEM. Significance was determined using one-way ANOVA with Tukey's post-hoc analysis **** $p < 0.0001$).

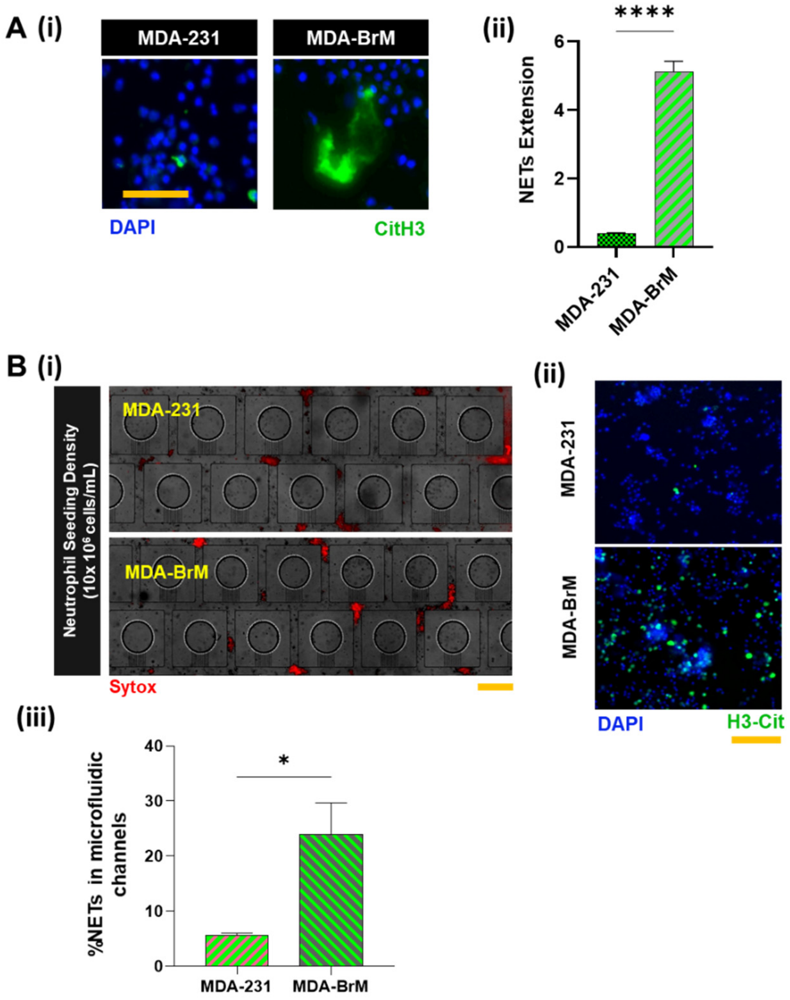

Figure S3: A (i) Representative images of the neutrophils (stained blue) incubated in tumor conditioned media and immunostained for citH3 (green) after 24 hours to look for NETs. Scale bar represents 50 µm (ii) NETs produced by neutrophils in MDA-BrM - CM showed a significantly greater spread (p<0.0001) than MDA-231. **B** (i) Representative images of Neutrophils in the microfluidic chemotaxis device were stained with Sytox (orange) to observe for NETs . Scale bar represents 200 µm (ii) The cells were washed carefully from the channel and collected in a well plate, and immunostained for citH3 (green). Scale bar represents 100 µm (iii) Neutrophils collected from the MDA-BrM chemotaxis device showed significantly greater (p<0.05) citH3 /+ signaling than MDA-231. (All data collected from at least $n = 3$ repeats of N = 3 experiments, mean ± SEM. Significance was determined using one-way ANOVA with Tukey's post-hoc analysis * $p < 0.05$, **** $p < 0.0001$).

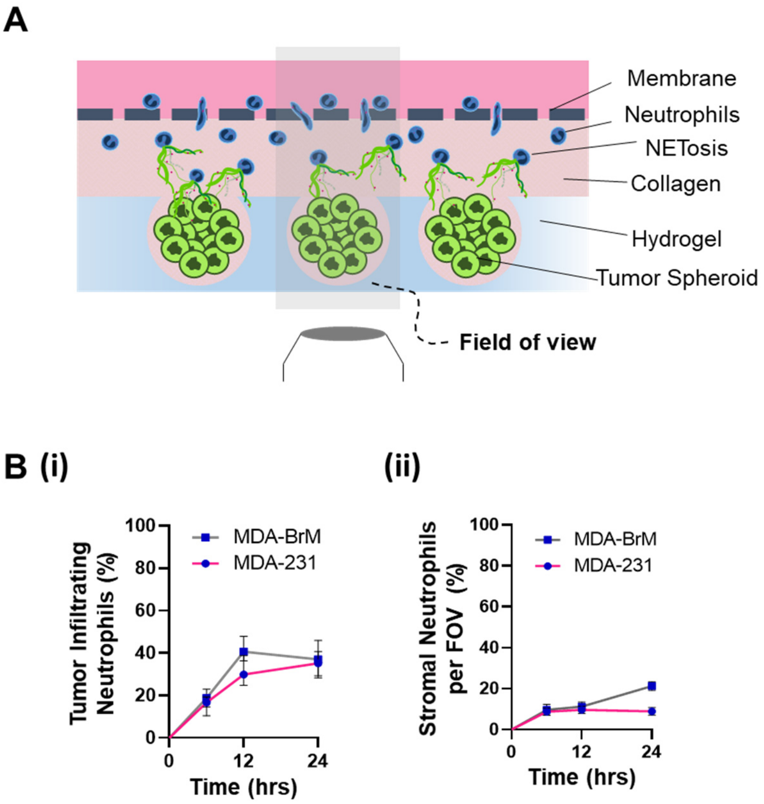

Figure S4: **A**. Schematic representation of the in vivo like neutrophil-tumor interactions within a Tumor-Immune Microenvironment-on-Chip (TIME-on-Chip) [12]. Tumor-Associated Neutrophils extravasate across the porous membrane into the tumor tissue (collagen), generate tumor-aiding Neutrophil Extracellular Traps (NETs), that could stimulate tumor invasion into the stromal region. **B**. Count of the total number of intact neutrophils per field of View, that infiltrated into the MDA-231 and MDA-BrM tumors over 24 hours (i) cells present on the spheroids i.e tumor-contacted and (ii) cells accumulating around the spheroids in the peripheral collagen region.

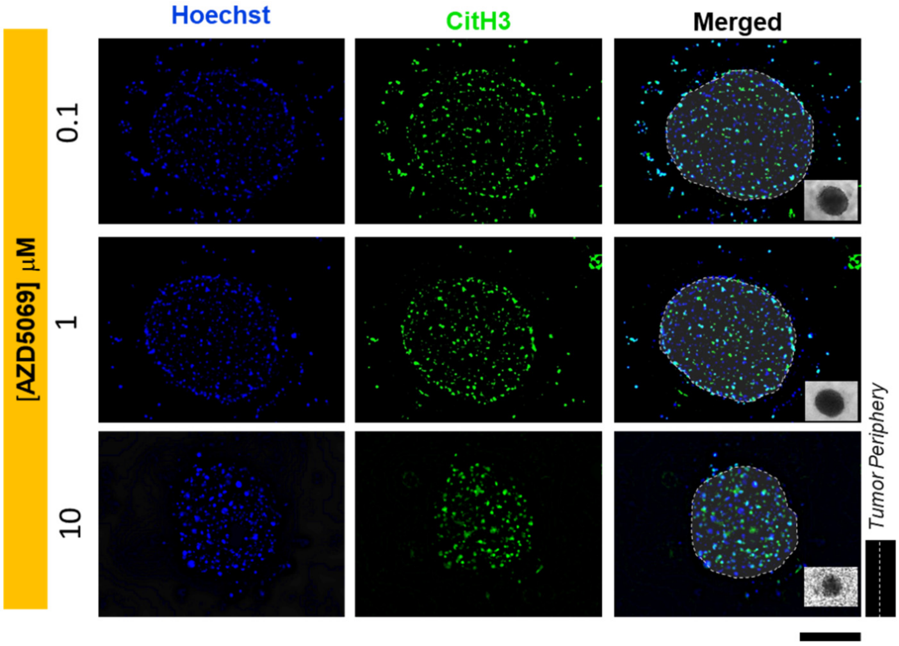

Figure S5: Representative images of the neutrophil infiltration into BrM tumors and the effect of pre-treatment with different concentrations of AZD5069, a small molecule CXCR2 antagonist, in reducing both neutrophil (Hoechst blue) influx and NETs formation (identified by immunostaining for CitH3 green). Scale bar represents 100 μm.

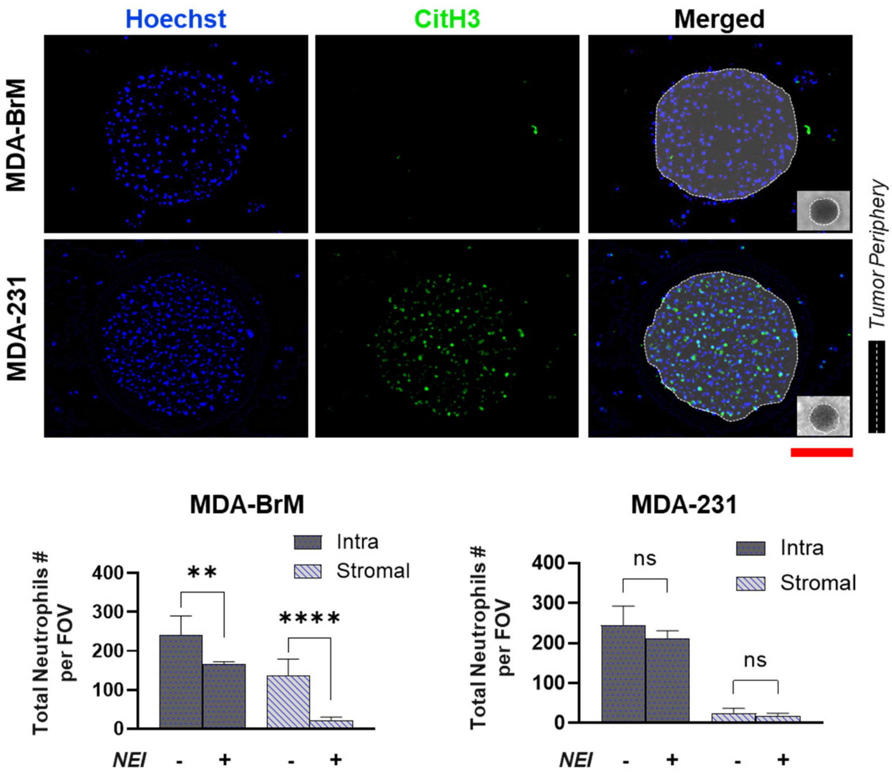

Figure S6: Representative images of neutrophils pre-treated with Sivelestat, a Neutrophil Elastase Inhibitor, infiltrating into the MDA-BrM and MDA-231. Upon NEI treatment whereas significant decrease was observed in neutrophil infiltration into the MDA-BrM for both, intraspheroidal and stromal accumulation. Whereas, NEI treatment had no effect on neutrophil infiltration into MDA-231 Scale bar represents 100 μm. (For total n = 9 spheroids measured over 3 experiments, mean ± SEM, t test, ** $p < 0.005$, **** $p < 0.0001$, ns = not significant).

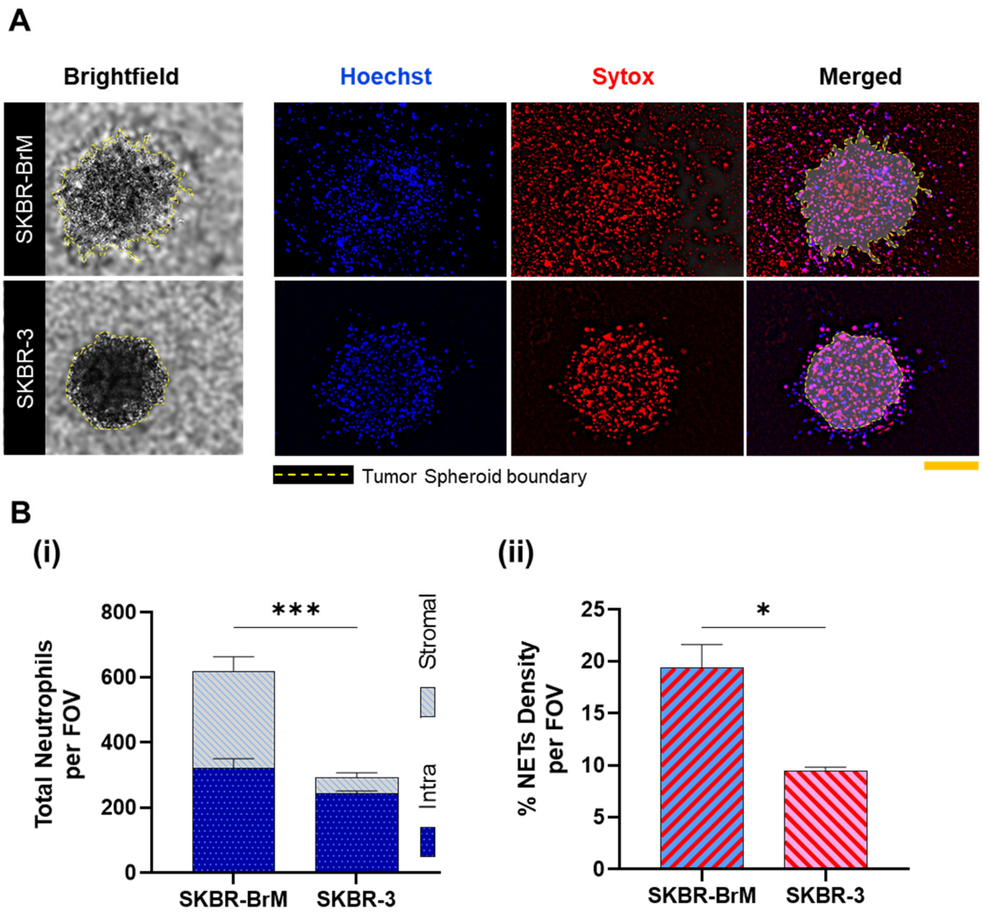

Figure S7: **A**. Representative images of the HER2+ breast tumor spheroids generated with the SKBR-3 cell line, and their brain metastatic variants showing the infiltration of neutrophils (stained Hoechst blue) and the NETs generated (stained with DNA marker sytox red) within the tumor niche. Scale bar represents 100 µm. **B.** (i) A count of the total intact neutrophils that infiltrated into the respective tumors, quantified within the field of view. Neutrophils that migrated toward the SKBR-3 brain metastatic tumors, after 24 hours was significantly greater ($p < 0.001$) than observed with SKBR-3. (ii) The total NETs density per field of view was significantly higher ($p < 0.05$) in SKBR-BrM than SKBR-3. (for total n = 9 spheroids measured over 3 experiments, mean ± SEM, t test, * $p < 0.05$, *** $p < 0.001$)).
